# CT image-based machine learning models for predicting blood eosinophil levels in acute exacerbation of chronic obstructive pulmonary disease

**DOI:** 10.1186/s12890-026-04219-w

**Published:** 2026-03-10

**Authors:** Shuiqing Zhao, Yanan Wu, Lirong Du, Hui Jia, Patrice Monkam, Wei Qian, Ruiying Wang, Shuyue Xia, Shouliang Qi

**Affiliations:** 1 College of Medicine and Biological Information Engineering, Northeastern University, Shenyang, China; 2 Key Laboratory of Intelligent Computing in Medical Image, Ministry of Education, Northeastern University, Shenyang, China; 3 School of Health Management, China Medical University, Shenyang, China; 4 Department of Respiratory and Critical Care Medicine, Shanxi Bethune Hospital, Shanxi Academy of Medical Sciences, Tongji Shanxi Hospital, Third Hospital of Shanxi Medical University, Taiyuan, China; 5 Department of Respiratory and Critical Care Medicine, The Affiliated Center Hospital of Shenyang Medical College, Shenyang, China

**Keywords:** Machine learning, Radiomics, Computed tomography, Acute exacerbation of chronic obstructive pulmonary disease, Blood eosinophils

## Abstract

**Background:**

Acute exacerbation of chronic obstructive pulmonary disease (AECOPD) is characterized by a significant worsening of respiratory symptoms. Blood eosinophil levels are a key predictor of glucocorticoid efficacy in AECOPD patients; however, their stability can present challenges. Predicting stable eosinophil levels from CT images is essential for optimal patient management.

**Methods:**

This study utilized CT images from 482 AECOPD patients across two hospitals. Dataset 1 comprised 193 patients for model development, while Dataset 2 included 289 patients for external validation. A threshold of 2% eosinophil was used to differentiate between high and low eosinophil levels. A machine learning model was developed to predict eosinophil levels using CT radiomics and quantitative computed tomography (QCT) features. Radiomics features were extracted, and feature selection was performed using random forest (RF) algorithms. Segmentation of pulmonary lobes, airways, and blood vessels yielded 20 QCT features. A Gradient Boosting (GB) classifier was then trained on the fused features.

**Results:**

The GB classifier with radiomics features demonstrated strong performance, achieving an accuracy (ACC) of 0.734 and an area under the curve (AUC) of 0.838 on the test set of Dataset 1. In external validation, the ACC and AUC were 0.624 and 0.671, respectively. After fusing QCT features, the ACC and AUC improved to 0.786 and 0.843, respectively, with external validation results of 0.673 and 0.697.

**Conclusion:**

The CT image-based machine learning model can predict blood eosinophil levels in AECOPD patients, providing a noninvasive and stable assessment. It has potential for future clinical application following further validation and external testing.

## Background

Chronic obstructive pulmonary disease (COPD) is a prevalent respiratory condition that poses a significant global public health challenge due to its morbidity and high mortality rates [1, 2]. Acute exacerbations and associated comorbidities are significant contributors to the mortality of many COPD patients [3, 4]. Blood eosinophil is a biomarker to monitor COPD progression among these factors. Numerous studies have demonstrated that eosinophilia can influence the clinical outcomes of acute exacerbations of COPD (AECOPD) [5, 6].

Blood eosinophils indicate eosinophilic inflammation in the airways and predict the response to inhaled corticosteroid (ICS) therapy [7, 8]. Martin et al. [9] found that the low blood eosinophil counts in AECOPD patients hospitalized are associated with prolonged hospitalization. Most AECOPD studies use 200 cells/mL and/or 2% as thresholds to identify eosinophil levels [10, 11]. Pasco et al. [12] found that the risk of moderate-to-severe AECOPD increased as eosinophil levels rose, mainly when peripheral blood eosinophil levels were ≥ 1.7%. Conversely, Hong et al. [13] noted that higher blood eosinophil counts were associated with a more rapid decline in lung function. According to the 2024 GOLD guidelines, blood eosinophil counts can predict the effectiveness of ICS in preventing future exacerbations [14].

In clinical practice, blood eosinophil counts are typically obtained through routine blood tests. Relevant studies have demonstrated that blood eosinophil counts can be influenced by various factors, including age, gender, atopy, and environmental exposure [15–18]. Significant advances in computed tomography (CT) technology, particularly high-resolution computed tomography (HRCT), which provides more precise and detailed images than conventional CT, have become increasingly crucial for assessing COPD. Developing a fast, accurate, and reliable method for automatically predicting eosinophil levels based on CT images is essential for enhancing the intervention and treatment of COPD patients. This approach provides physicians with targeted intervention recommendations, ensuring timely and appropriate patient care and, to some extent, alleviating the workload of healthcare providers.

Radiomics features in lung disease imaging are regarded as one of the cutting-edge tools in medicine [19]. Previous studies have shown that imaging histology can be applied to diagnosing and predicting COPD and related diseases. Lin et al. [20] assessed cardiovascular disease (CVD) risk in COPD patients using CT-based whole lung radiomics. Zhao et al. [21] proposed a method for staging COPD severity by combining lobe radiomics features. Zhou et al. [22] utilized whole lung radiomics to distinguish between healthy individuals and COPD patients. Amudala et al. [23] evaluated the performance of standard-dose and low-dose CT radiomics features in diagnosing COPD. The studies above indicate that radiomics features are crucial for the diagnosis and treatment prediction of COPD.

In COPD, quantitative computed tomography (QCT) has shown a significant correlation with disease progression and mortality [24–26]. Zhang et al. [27] proposed a method for the early diagnosis of high-risk COPD patients using QCT. Konietzk et al. [28] employed QCT to monitor the progression of emphysema in COPD patients at three-month intervals. Moslemi et al. [29] utilized QCT and CT radiomics to predict the emergency department (ED) visits rate and the length of hospital stays. In summary, while QCT and CT radiomics have shown the potential for predictive diagnosis in COPD, few studies have concentrated on predicting eosinophil levels in AECOPD patients using CT images.

In this study, we propose a method for predicting eosinophil levels in AECOPD patients based on CT images. Specifically, the study employed CT radiomics and QCT features to predict eosinophil count levels. The combination of CT radiomics and QCT results in a more comprehensive and enriched characterization, which enhances the predictive model’s performance.

The contributions of this paper are as follows:

- A CT image-based machine learning model is proposed to predict eosinophil levels in AECOPD, addressing the instability of measuring the eosinophil index by routine blood tests.
- CT radiomics and QCT features are integrated to improve the performance of predicting eosinophil levels in AECOPD patients.
- Two central datasets are utilized to demonstrate the feasibility and generalization ability of predicting eosinophil levels in AECOPD using CT images.

## Methods

### Datasets

- *Dataset 1*: The dataset comprised 193 AECOPD patients screened at the Affiliated Central Hospital of Shenyang Medical College, with slice thicknesses ranging from approximately 0.5 to 1.2 mm. The inclusion criteria were as follows: (1) Complete CT images are available, allowing for all QCT measurements to be analyzed; (2) No QCT measurements have any missing values; (3) Complete demographic information and clinical indicators are provided; (4) All the factors which might affect the measurement of eosinophil level through routine blood tests have been excluded, including diurnal variation, recent use of corticosteroids or antibiotics, allergic reactions, parasitic infections. Based on the eosinophil count at the time of admission, the enrolled AECOPD patients were categorized into two groups: 87 cases in the high eosinophil level group (eosinophil% ≥2%) and 106 cases in the low eosinophil level group (eosinophil% <2%). Table 1 presents the demographic information and clinical characteristics of the 193 AECOPD patients.
- *Dataset 2*: The dataset was obtained from Shanxi Bethune Hospital and comprises 289 CT scans of AECOPD patients, including 115 patients in the high eosinophil group and 174 patients in the low eosinophil group. This dataset was used as an external validation set to further assess the prediction model’s predictive capability for eosinophil level in AECOPD patients. Table 2 presents the patient’s demographic information and clinical characteristics in the external validation set.

**Table 1:** Demographic information and clinical characteristics of patients in Dataset 1 (EOS demonstrates eosinophil)

Clinical information	High EOS group	Low EOS group	*P* value*
Case	87	106	-
Sex (male/female)	56/31	64/42	0.57
Age (years)	71.34 ± 11.08	72.40 ± 10.05	0.49
Body mass index (BMI-kg/m2)	23.50 ± 3.64	22.49 ± 1.14	0.08
Smoking years	22.95 ± 21.46	25.92 ± 22.29	0.35
Length of hospitalization (days)	9.72 ± 3.87	10.90 ± 5.45	0.09

* Two-sample t-test

**Table 2:** Demographic information and clinical characteristics of patients in Dataset 2 (EOS demonstrates eosinophil)

Clinical information	High EOS group	Low EOS group	*P* value*
Case	115	174	-
Sex (male/female)	95/20	141/33	0.84
Age (years)	67.20 ± 8.77	68.56 ± 8.46	0.15
Smoking years	23.2 ± 16.31	29.4 ± 15.69	0.01
Length of hospitalization (days)	6.93 ± 3.23	8.72 ± 4.04	0.01

* Two-sample t-test

Table 3 compares the technical parameters of CT scans from two medical imaging datasets (Dataset1 and Dataset2) with the following information:

**Table 3:** CT acquisition protocols of two cohorts

Cohorts	Scanning equipment	Tube voltage (kV)	Pitch	Thickness (mm)	Matrix	Type
Dataset1	NeuViz 128 1.0	120	1	0.5–1.2	512 × 512	Standard-dose
Dataset2	SIEMENS syngo CT 2012B	120	1.35	1	512 × 512	Standard-dose

### Overall framework

**Fig. 1:**
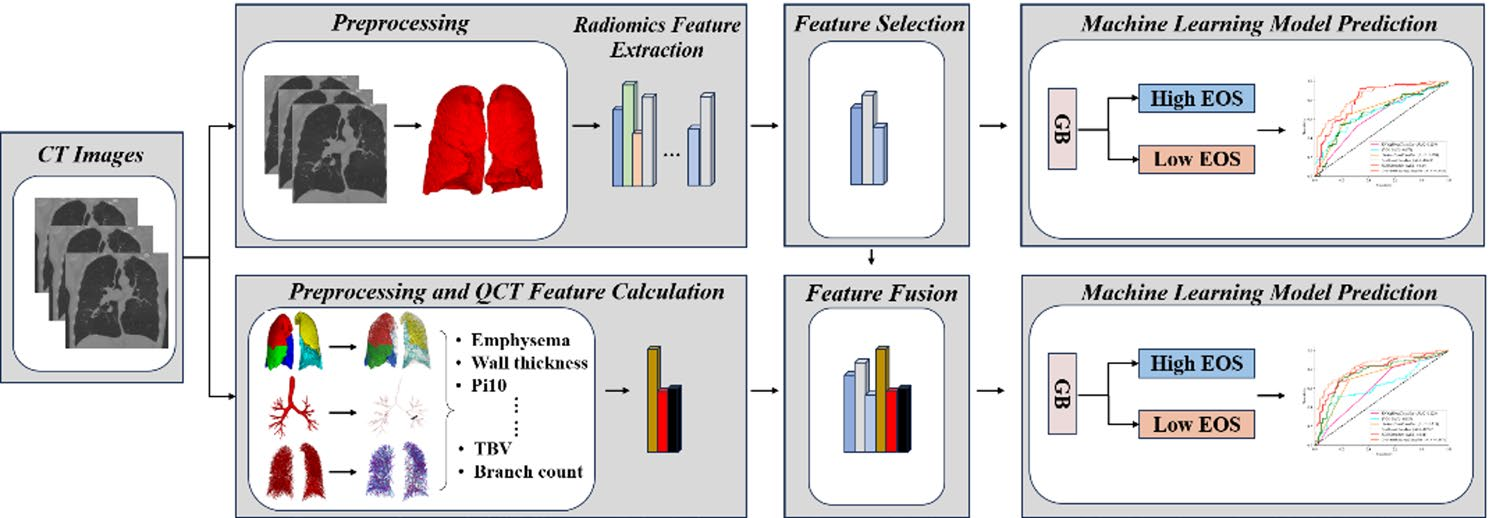
The framework for blood eosinophil levels prediction using CT images

Figure 1 illustrates the workflow of this study’s proposed CT image-based prediction method for blood eosinophil levels. First, the lung region is segmented from CT images by the lungmask [30], and the lobes, airway, and blood vessels are segmented by the proposed method [31–33]. All segmenation will be visually inspected by two experienced radiologists to ensure anatomical accuracy and consistency of segmentation results. Second, the same as other studies [22, 34, 35], for small ROI, the features are sensitive to the segmentation methods. However, lung filed is very large, the influence is very slight. CT image radiomics features are extracted from the lung region, while in-house methods extract QCT features. Next, the radiomics features are selected using the random forest (RF) algorithm, and feature fusion is conducted with the QCT features. Finally, a Gradient Boosting (GB) classifier is developed to predict blood eosinophil levels.

### Procedure for radiomics model

#### Preprocessing

According to the proposed method [30], automatic lung segmentation from CT images is performed. Before CT radiomics feature extraction, the CT images are resampled to $1mm\times1mm\times1mm$ to reduce the effect of different layer thicknesses and the interference of noise. The bin width is set 20.

#### Radiomics feature extraction

Pyradiomics [36] is a tool to extract features from raw or image-processed CT images. With Pyradiomics, feature extraction and analysis can be easily performed to support further research and applications in radionics. Radiomics features can be categorized explicitly into the following three types: first-order statistics, texture features, and higher-order filter transform features. Definitions and detailed explanations of radiomics features can be found in the Pyradiomics documentation.

#### Feature selection

Feature selection helps to improve the performance of predictive models by identifying and retaining the most relevant and informative features, reducing the risk of overfitting, and reducing dimensionality by eliminating irrelevant or redundant variables. The RF [37] is used for feature selection. Specifically, the absolute value of the importance coefficient of each feature is first ranked to reflect the degree of influence of the feature on the classification effect of the model and to analyze the importance of each feature. According the recommendation by Gillies et al. [38], about 10 samples or patients are required for each feature in a binary classifier. We have 193 patients and 20 features, which approximately according to the recommended requirements. Thus, the top 20 ranked features are taken as the result of feature selection. By randomly selecting features for each tree split, the model reduces the dominance of highly correlated features and helps to better identify informative variables. Although Random Forest does not eliminate multicollinearity, it is less sensitive to it compared to linear models. And the model we use in the training is not linear.

#### Machine learning model prediction

The GB is the prediction model [39]. The GB is a supervised machine learning algorithm known for its effectiveness in various classification tasks. It can handle data with nonlinear and high-dimensional features and has been widely applied across multiple fields.

To evaluate the proposed method’s reliability and generalizability, the Dataset1 were divided into a training set (135 samples: 74 patients in the low EOS group and 61 in the high EOS group) and an internal test set (58 samples: 32 patients in the low EOS group and 26 in the high EOS group). The training set was employed for feature selection and training the model, while performance testing was carried out separately on the internal validation set to ensure experimental accuracy. Dataset 2 was used as an external validation set.

### Procedure for fusion model

#### Preprocessing and QCT feature calculation

According to the proposed method [31–33], the lobes, airways, and blood vessels were automatically segmented. The in-house scientific software (Key Laboratory of Intelligent Computing for Medical Imaging, Northeastern University, Shenyang, Liaoning, China) was used to quantify emphysema, airways and blood vessels. Table 4 presents a comprehensive list of QCT measurements utilized in this study and their definitions. (1) Emphysema measurements: The emphysema index [40] is crucial for quantifying the degree of emphysema. The percentage of low attenuation area with attenuation less than − 950 HU (%LAA-950) for the entire lung as well as for each lobe, including the right upper lobe (RUL), right middle lobe (RML), and right lower lobe (RLL), the right lung (RL), as well as the left upper lobe (LUL), left lower lobe (LLL) and left lung (LL). (2) Airway measurements: The airway measurements focus on the structural characteristics of the airways, offering valuable insights into their morphology and potential pathological changes. Wall thickness (WT) [41]: This measure assesses the thickness of the airway walls, which may indicate inflammatory processes or structural remodeling associated with various lung diseases. Wall area percentage (WA%) [42]: This percentage reflects the proportion of the airway cross-section occupied by the wall, providing insights into airway calibre and potential obstruction. Pi10 [43]: This index estimates the wall thickness of an airway with an ideal internal circumference of 10 mm, which is particularly important for assessing small airway disease. (3) Blood vessel measurements: The vessel measurements encompass a range of quantitative measures computed using the VesselVio [44], which provides a detailed analysis of the pulmonary vasculature. The following indices include the aggregate vessel volume for vessels less than 5 mm^2^ (BV5), network length, surface area, branch points, endpoints, number of segments, mean segment radius, mean segment length, and mean segment tortuosity.

**Table 4:** The definitions of 20 QCT features

No.	QCT feature	Description	Category
1	RUL emphysema index	%LAA-950 in the right upper lobe	Emphysema
2	RML emphysema index	%LAA-950 in the right middle lobe	
3	RLL emphysema index	%LAA-950 in the right lower lobe	
4	RL emphysema index	%LAA-950 in the right lung	
5	LUL emphysema index	%LAA-950 in the left upper lobe	
6	LLL emphysema index	%LAA-950 in the left lower lobe	
7	LL emphysema index	%LAA-950 in the left lung	
8	All lung emphysema index	%LAA-950 in the whole lung	
9	Pi10	An estimate of the wall thickness of an airway with an ideal internal circumference of 10 mm.	Airway
10	WA%	Percentage of airway wall area	
11	WWT	Mean value of airway wall thickness	
12	BV5	The aggregate vessel volume for vessels less than 5 mm^2^	Blood vessel
13	Vessel network length	Total vessel tree length	
14	Vessel surface area	Total vessel surface area	
15	Vessel branch points	Total number of vessel branch	
16	Vessel endpoints	Total number of end branch	
17	Number of segments	Total number of identifiable vessel segments in the vascular network	
18	Mean segment radius	Mean value of vessel segment radius	
19	Mean segment length	Mean value of vessel segment length	
20	Mean segment tortuosity	Mean value of vessel segment tortuosity	

#### Feature fusion

Feature fusion combines multiple features to obtain more prosperous and effective input features and improves the performance of machine learning models. For splicing, 20 CT radionics and 20 QCT features are used.

#### Machine learning model prediction

The methods are as same as those in the radiomics model.

### Implementation details and evaluation metrics

Four evaluation metrics are used to assess eosinophil level prediction performance, including accuracy (ACC), sensitivity (SEN), positive predictive value (PPV), area under curve (AUC), receiver operating characteristic (ROC), The formulas are as follows:

1 $$ACC=\frac{TP+TN}{TP+TN+FP+FN}$$

2 $$SEN=\frac{TP}{TP+FN}$$

3 $$PPV=\frac{TP}{TP+FP}$$

where true positive ($TP$) denotes the number of patients correctly predicted high eosinophil, true negative ($TN$) denotes the number of patients correctly predicted low eosinophil, false positive ($FP$) denotes the number of patients which is low eosinophil mispredicted high eosinophil, false negative ($FN$) denotes the number of patients which is high eosinophil mispredicted low eosinophil.

The ROC curve is represented by the false positive rate on the x-axis and the true positive rate on the y-axis [45]. The closer the ROC curve is to the upper left corner, the larger the AUC and the better the classification model performance [46].

In this study, other machine learning models are used, including K-nearest neighbour (KNN) [47], support vector machine (SVM) [48], decision tree [49], AdaBoost [50], and XGBoost [51]. Classifiers use their default parameter settings to facilitate more direct comparisons between models and highlight each algorithm’s out-of-the-box functionality. Each classifier is independent and has no relationship with other classifiers and no information leakage. There is no need for multiple comparisons or corrections. In machine learning or deep tilting research [21, 52], we almost always perform comparisons between different models in this way.

## Results

### Prediction performance of radiomics model with different numbers of features and feature selection methods

In Table 5, we analyze the impact of varying the number of features selected on the performance of the RF model for predicting eosinophil levels. With 15 selected features, the RF model achieves an ACC of 0.721 and SEN, PPV and AUC scores of 0.713, 0.724, and 0.800, respectively. However, when the number of selected features is increased to 25, there is a slight decline in performance, resulting in an ACC of 0.716, with SEN, PPV, and AUC scores of 0.708, 0.725, and 0.821. Based on these findings, the best overall performance is achieved when the feature selected number is set to 20, yielding an ACC of 0.734 and SEN, PPV, and AUC scores of 0.732, 0.752, and 0.838.

**Table 5:** Performance comparison using different numbers of radiomics features on Dataset 1

Feature selections number	ACC	SEN	PPV	AUC
15	0.721	0.713	0.724	0.800
20	**0.734**	**0.732**	**0.752**	**0.838**
25	0.716	0.708	0.725	0.821

Note: Bold values highlight the maximum values

**Fig. 2:**
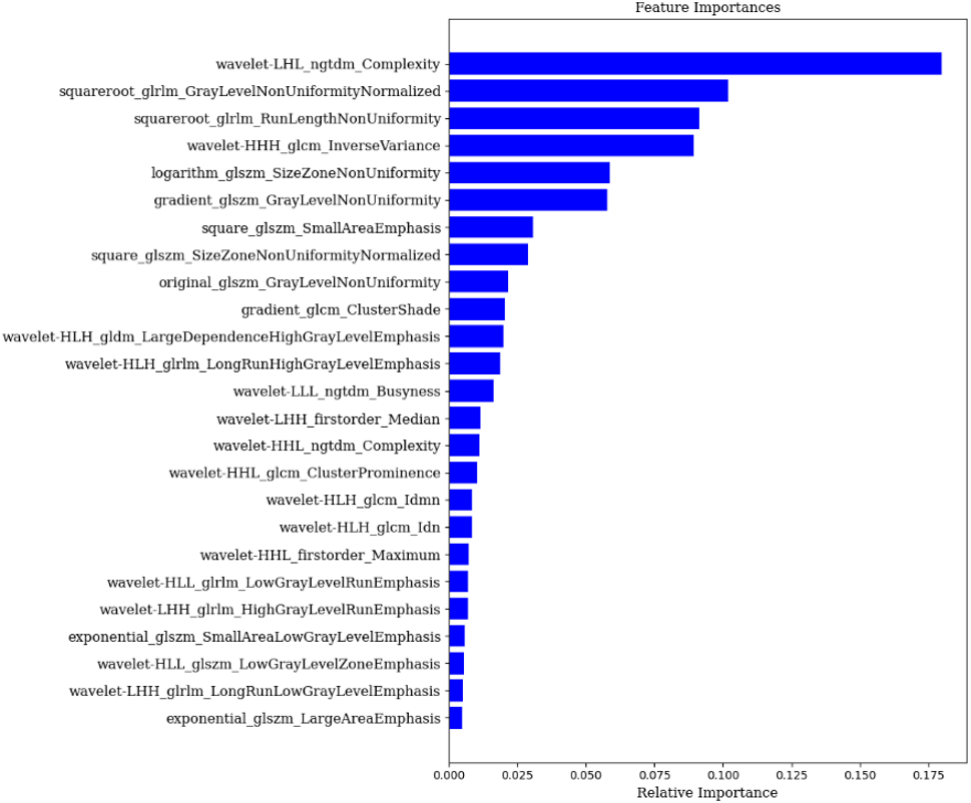
20 Radiomics features and corresponding coefficients after dimensionality reduction by RF

Figure 2 illustrates the top 20 essential features of the screening, with wavelet transform features and the Gray-Level Co-Occurrence Matrix showing high importance. The features quantify the spatial relationship between pixel grey values and texture properties and are more suitable for eosinophil prediction.

Table 6 compares the LASSO and RF methods for feature selection. The LASSO method achieves an ACC of 0.676, with corresponding SEN, PPV, and AUC scores of 0.681, 0.684, and 0.725. In contrast, the RF method exhibits significantly better performance. The results indicate that the RF approach is more effective for predicting eosinophil levels.

Overall, these analyses highlight the importance of feature selection in enhancing predictive ACC, with RF and a feature selection number of 20 emerging as the more robust method.

**Table 6:** Performance comparison using different feature selection methods on Dataset 1

Method	ACC	SEN	PPV	AUC
Lasso	0.676	0.684	0.681	0.725
RF	**0.734**	**0.752**	**0.732**	**0.838**

Note: Bold values highlight the maximum values

### Prediction performance of models with different features

Table 7 presents the results of predicting blood eosinophil levels using different features with the GB method on Dataset 1. The model was trained using three distinct feature combinations: CT radionics, QCT features, and a fusion of both. Specifically, the CT radiomics-based model achieves an ACC of 0.734, with SEN, PPV, and AUC around 0.732, 0.752, and 0.838. In contrast, for the QCT-based model, ACC, SEN, PPV, and AUC are 0.602, 0.594, 0.603, and 0.590.

However, combining both features significantly improved performance, resulting in an ACC of 0.786, with corresponding SEN, PPV, and AUC scores of 0.778, 0.786, and 0.843. Overall, these results indicate that eosinophil levels can be effectively predicted using CT radiomics features, and further enhancement in predictive ACC can be achieved by fusing QCT features.

**Table 7:** Performance comparison using different features on Dataset 1

CT radiomics	QCT	ACC	SEN	PPV	AUC
√		0.734	0.732	0.752	0.838
	√	0.602	0.594	0.603	0.590
√	√	**0.786**	**0.778**	**0.786**	**0.843**

Note: Bold values highlight the maximum values

### Prediction performance of models with different machine learning methods

Table 8 comprehensively evaluates machine learning methods for predicting blood eosinophil levels on Dataset 1 (training dataset). The task is to predict high and low blood eosinophil levels. The machine learning methods include KNN, SVM, Decision Tree, AdaBoost, XGBoost, and Gradient Boosting. 

**Table 8:** Performance comparison of models with six machine learning methods on Dataset 1

Features	Machine learning method	ACC	SEN	PPV	AUC
CT radiomics	KNN	0.612	0.613	0.615	0.624
	SVM	0.653	0.645	0.654	0.671
	Decision Tree	0.706	0.703	0.705	0.699
	AdaBoost	0.584	0.583	0.586	0.671
	XGBoost	0.703	0.704	0.704	0.814
	GB	**0.734**	**0.732**	**0.752**	**0.838**
CT radiomics and QCT	KNN	0.585	0.576	0.578	0.623
	SVM	0.652	0.644	0.648	0.629
	Decision Tree	0.724	0.723	0.725	0.719
	AdaBoost	0.756	0.744	0.757	0.764
	XGBoost	0.733	0.732	0.734	0.801
	GB	**0.786**	**0.778**	**0.786**	**0.843**

Note: Bold values highlight the maximum values

For the CT radiomics model, the performance metrics of each method are as follows: KNN achieves an ACC of 0.612, SEN of 0.613, PPV of 0.615, and AUC of 0.624. The SVM method shows slightly better results with an ACC of 0.653, while the Decision Tree reaches an ACC of 0.706. AdaBoost performs poorly, with an ACC of 0.584. The XGBoost method performs better with an ACC of 0.703. The GB method achieved the highest metrics: an ACC of 0.734, SEN of 0.732, PPV of 0.752, and AUC of 0.838. This highlights the effectiveness of the GB method for CT radiomic features.

Most methods show improved performance for CT radiomics and QCT model. KNN’s performance drops slightly, with an ACC of 0.585, while SVM maintains an ACC of 0.652. The Decision Tree method also performs well, achieving an ACC of 0.724. Notably, the AdaBoost method improves significantly, achieving an ACC of 0.756. The XGBoost method improves slightly with an ACC of 0.733. However, the GB method again demonstrates the best performance, reaching an ACC of 0.786, SEN of 0.778, PPV of 0.786, and AUC of 0.843. This improvement highlights the fusion of QCT features, likely providing additional relevant information that enhances the method’s predictive capabilities.

**Fig. 3:**
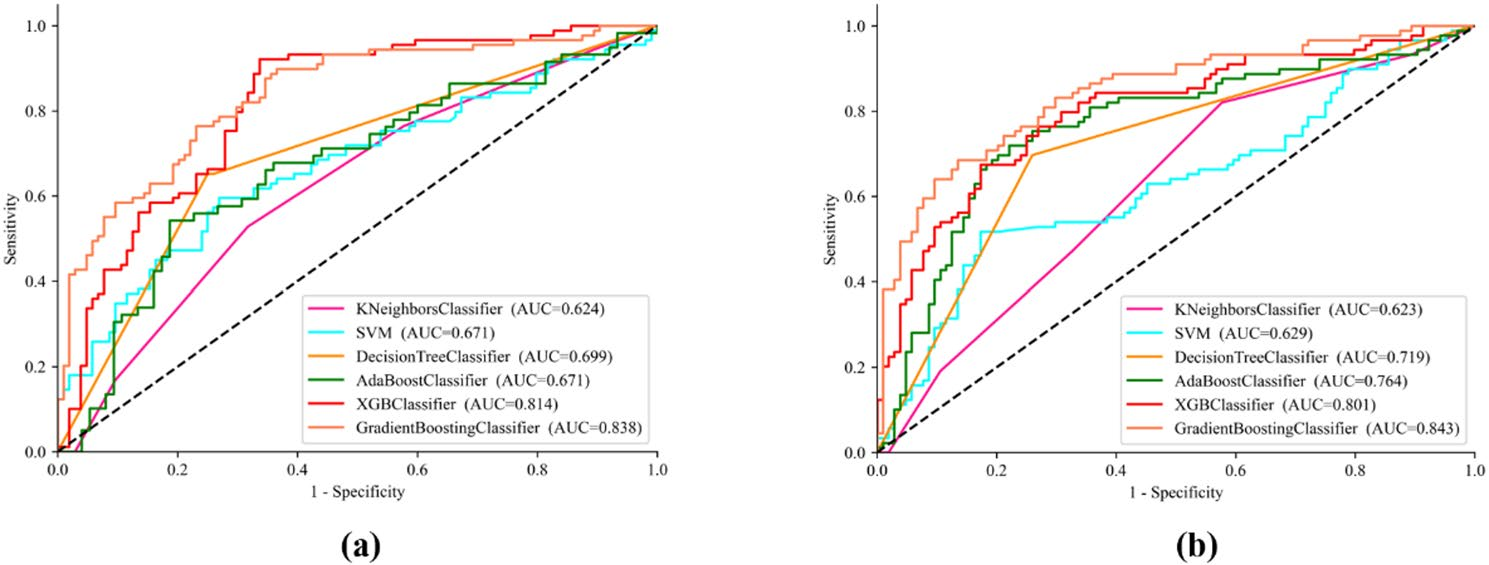
ROC curves for blood eosinophil levels prediction for different models on Dataset 1. (**a**) With CT radiomics features; (**b**) With CT radiomics and QCT features

Additionally, as shown in Figs. 3(a) and (b), the evaluation of blood eosinophil level predictions for each method is illustrated through ROC curve analysis, allowing for a visual representation of each method’s performance. The GB method achieves the best results across two feature extraction methods, demonstrating its robustness and effectiveness.

These findings indicate that the Gradient Boosting method effectively predicts blood eosinophil levels using CT radiomics and significantly enhances performance when combined with QCT features. The results suggest that further research into hybrid features and method optimization could yield better outcomes for predicting blood eosinophil levels.

### Prediction performance on external validation Dataset 2

**Fig. 4:**
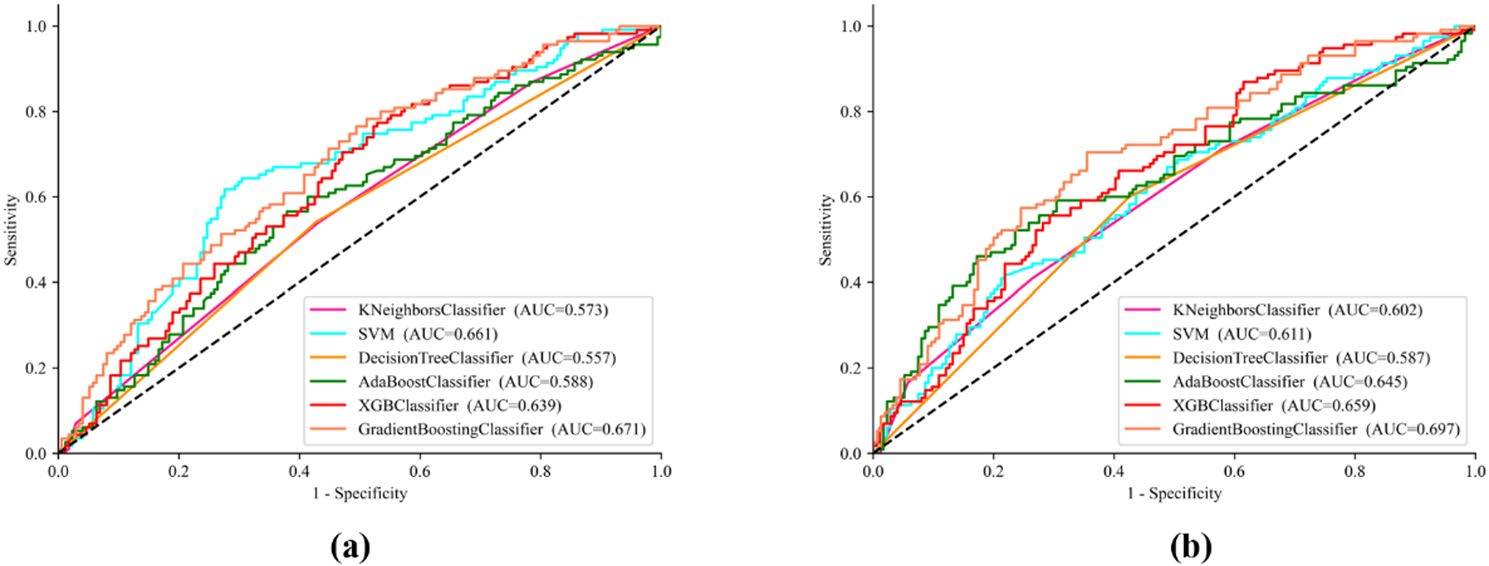
ROC curves for blood eosinophil levels prediction for different models on Dataset 2. (**a**) With CT radiomics features; (**b**) With CT radiomics and QCT features

As shown in Table 9; Fig. 4(a), and (b), to further validate the method’s reliability, we extend the proposed method to an external validation set (Dataset 2). Dataset 2 is tested on a previously trained model. Utilizing multiple machine learning methods, blood eosinophil levels prediction is performed on Dataset 2 with the same features.

**Table 9:** Performance comparison of models with six machine learning methods on Dataset 2

Features	Machine learning method	ACC	SEN	PPV	AUC
CT radiomics	KNN	0.602	0.534	0.556	0.573
	SVM	0.613	0.583	0.603	0.661
	Decision Tree	0.562	0.560	0.558	0.557
	AdaBoost	0.583	0.584	0.586	0.588
	XGBoost	0.592	0.603	0.594	0.639
	GB	**0.624**	**0.617**	**0.619**	**0.671**
CT radiomics and QCT	KNN	0.614	0.572	0.583	0.602
	SVM	0.634	0.603	0.615	0.611
	Decision Tree	0.586	0.597	0.582	0.587
	AdaBoost	0.634	0.623	0.627	0.645
	XGBoost	0.643	0.616	0.627	0.659
	GB	**0.673**	**0.667**	**0.669**	**0.697**

Note: Bold values highlight the maximum values

For the CT radiomics method, KNN achieves an ACC of 0.602, SEN of 0.534, PPV of 0.556, and AUC of 0.573, while SVM reaches an ACC of 0.613. The Decision Tree shows lower performance with an ACC of 0.562. AdaBoost and XGBoost also demonstrate limited effectiveness, with ACCs of 0.583 and 0.592, respectively. In contrast, the GB method performs better than others in this method, achieving an ACC of 0.624, SEN of 0.617, PPV of 0.619, and AUC of 0.671.

For the CT radiomics with the QCT features method, the KNN method maintains an ACC of 0.614 and SEN of 0.572. The SVM method shows slightly improved results with an ACC of 0.634. The Decision Tree again performs modestly with an ACC of 0.586, while AdaBoost and XGBoost reach an ACC of 0.634 and 0.643. Notably, the GB method excels in this combined feature set, achieving the highest ACC of 0.673, SEN of 0.667, PPV of 0.669, and AUC of 0.697.

It is important to note that while training is conducted on Dataset 1, external validation occurs on Dataset 2, which is collected using a different device and from a different geographic region. This variation in the data acquisition process may explain the observed decrease in performance when applying the model to Dataset 2.

Overall, these findings reinforce the efficacy of the GB method for predicting blood eosinophil levels, even when validated on an external dataset. The results also highlight the importance of feature selection and model robustness, suggesting that further investigation into improving generalizability across different devices and regions could enhance predictive performance.

## Discussion

This study proposes a new way to predict blood eosinophil levels based explicitly on CT radiomics and QCT. The combination of QCT provided a better overview when predicting blood eosinophil levels compare to the use of CT radiomics alone. The proposed method introduces a new direction for future blood eosinophil detection.

Previous studies have demonstrated the use of CT imaging to predict similar physiological indices. Geraghty et al. proposed multiple linear regression to predict an individual’s height, weight, body mass index, and body surface area with a single abdominal CT [53]. Park et al. developed a deep learning-based method for predicting lung function from low-dose CT images [54]. With the advancement of computer vision and machine learning techniques, the analysis of CT imaging has been significantly improved. Researchers have used algorithms to extract complex features from CT images to achieve more accurate predictions of physiological indicators. The combination of these techniques not only improves prediction ACC but also provides more substantial support for clinical decision-making. This also provides technical support and new ideas for this study.

Some studies have used blood eosinophil as a biomarker for ICS, suggesting that > 2% may be an appropriate threshold for the relative efficacy of ICS [55–57]. Meanwhile, blood eosinophils can potentially be a prognostic indicator in AECOPD [58], and there is an association between emphysema and blood eosinophil levels [59]. In conclusion, eosinophils not only have potential as a biomarker for assessing the efficacy of ICS but also show significant value in the prognostic assessment of AECOPD and studies related to emphysema. Routine blood counts make routine blood eosinophil measurements. Thus, we used 2% blood eosinophil (EOS) percentage as the cut-off threshold to construct a model predicting the classification of “high EOS (≥ 2%)” vs. “low EOS (< 2%)”. Thus, indirectly predicting the predicted eosinophil level.

The CT scans are typically performed when COPD patients require imaging for other clinical indications. Our model offers an “opportunistic” assessment of eosinophil levels using existing CT images, eliminating the need for additional tests or radiation exposure. Blood eosinophil levels can fluctuate due to various factors, leading to measurement instability. In contrast, CT imaging captures long-term pathological changes in the lungs, providing a more stable and representative assessment of eosinophilic inflammation in COPD. For patients needing frequent monitoring, noninvasive CT assessment may reduce the necessity for repeat blood draws, thereby alleviating patient discomfort and potential complications.

This is the first study to use CT images to predict blood eosinophil levels and the first to investigate using hybrid features to predict blood eosinophil levels. Blood eosinophil levels fluctuate throughout the day, leading to inconsistent readings based on the timing of blood draws. Factors such as stress, infection, recent corticosteroid use, or allergic reactions can temporarily alter eosinophil levels, affecting their reliability as stable biomarkers in certain clinical contexts. Our CT-based approach does not eliminate these external influences directly but aims to reduce measurement variability by offering a stable assessment of eosinophilic inflammation. Specifically, CT-based radiomics and quantitative CT features capture structural and pathological changes in the lungs, such as emphysema, airway remodeling, and vascular alterations. These changes are less affected by short-term physiological factors and instead reflect the cumulative effects of chronic eosinophilic inflammation, providing a more stable assessment that complements blood eosinophil measurements. Additionally, our approach posits that CT imaging features may capture chronic disease phenotypes linked to eosinophilic inflammation, potentially offering predictive insights into future eosinophil levels or treatment responses. This represents an advantage over isolated blood test results, which can vary significantly due to short-term factors. In conclusion, while we acknowledge that blood tests are the current gold standard for measuring eosinophil levels and that our methods were evaluated against these measurements, our study’s intent is not to replace blood tests but to provide a complementary tool for situations where blood eosinophil measurements are unreliable or unavailable.

In contrast, the model employing gradient boosting as a predictor has an ACC of 0.734 when relying exclusively on CT imaging features. However, in the external validation set, the ACC exceeds 0.624, which is the best performance among the selected classification models. Notably, when quantitative metrics are integrated into the model, the ACC improves significantly, reaching 0.786 on the training set and 0.673 on the external validation set. This improvement validates the model’s generalization ability and highlights the advantages of combining imaging features with CT quantitative metrics. Fusing these data types led to a partial improvement in predictive results, highlighting the importance of a multifaceted approach in predictive modeling.

Despite these encouraging findings, our study has some limitations. Firstly, sample size limitations are one of the main limitations of this study, and future work must include larger, multi-center datasets for external validation. Secondly, although the experiment was conducted on data from two centralized datasets, the effect was reduced in the external validation set (Dataset 2) due to equipment, region, and other factors. While the model showed good predictive performance on the training dataset (Dataset 1), further validation on more centralized datasets is needed to confirm its clinical utility. Thirdly, the performance of the radiomics approach across different data domains has not been thoroughly evaluated. Therefore, methods such as domain adaptation or other generative artificial intelligence techniques should to be considered in future studies [60–62]. Thirdly, the study focused on predicting blood eosinophils using CT imaging. Significant differences in smoking history and length of hospitalization were observed between the comparison groups in Data Set 2. However, confounding could not be fully corrected due to the lack of quantitative data on smoking pack-years and limitations in sample size. This residual confounding may affect the interpretation of outcomes, particularly for smoking-related endpoint indicators. Future prospective studies should prioritize obtaining refined exposure data to isolate true effects. And, this study did not include other clinical information or genetic data such as respiratory parameters, BMI, family history, and genetic factors. Finally, deep learning methods could be explored in future studies to predict blood eosinophil levels, as they have been successfully used in COPD identification, staging, and quantitation [63].

## Conclusion

This study demonstrates that CT image-based can provide a promising method for blood eosinophil levels prediction that is non-invasive and stable and, when further validated, can guide subsequent treatment regimens for COPD patients. In addition, CT radiomics features can provide global information. In contrast, quantitative CT can provide an intrinsic feature, and the fusion of CT radiomics and quantitative CT features can better enhance the prediction performance. This also demonstrates that there may be a link between macroscopic CT imaging phenotypes and inflammatory microphenotypes. Machine learning methods can identify this link and thus establish predictive relationships.

## Data Availability

No datasets were generated or analysed during the current study.

## Abbreviation

AECOPD: Acute exacerbation of chronic obstructive pulmonary disease
QCT: Quantitative computed tomography
RF: Random forest
GB: Gradient boosting
ACC: Accuracy
AUC: Area under the curve
COPD: Chronic obstructive pulmonary disease
ICS: Inhaled corticosteroid
CT: Computed tomography
HRCT: High-resolution computed tomography
CVD: Cardiovascular disease
EOS: Eosinophil
BMI: Body mass index
%LAA-950: The percentage of low attenuation area with attenuation less than − 950 HU
RUL: Right upper lobe
RML: Right middle lobe
RLL: Right lower lobe
RL: Right lung
LUL: Left upper lobe
LLL: Left lower lobe
LL: Left lung
WT: Wall thickness
WA%: Wall area percentage
BV5: The aggregate vessel volume for vessels less than 5 mm2
SEN: Sensitivity
PPV: Positive predictive value
ROC: Receiver operating characteristic
TP: True positive
TN: True negative
FP: False positive
FN: False negative
KNN: K-nearest neighbour
SVM: Support vector machine
